# Toxicity of Selected Monoterpenes and Essential Oils Rich in These Compounds

**DOI:** 10.3390/molecules27051716

**Published:** 2022-03-06

**Authors:** Karolina A. Wojtunik-Kulesza

**Affiliations:** Department of Inorganic Chemistry, Medical University of Lublin, Chodźki 4a, 20-093 Lublin, Poland; karolina.wojtunik@umlub.pl

**Keywords:** toxicity, monoterpenes, plant secondary metabolites, teratogenicity, neurotoxicity, abortifacient, genotoxicity

## Abstract

Monoterpenes make up the largest group of plant secondary metabolites. They can be found in numerous plants, among others, the *Lamiaceae* family. The compounds demonstrate antioxidative, antibacterial, sedative and anti-inflammatory activity, hence, they are often employed in medicine and pharmaceuticals. Additionally, their fragrant character is often made use of, notably in the food and cosmetic industries. Nevertheless, long-lasting studies have revealed their toxic properties. This fact has led to a detailed analysis of the compounds towards their side effects on the human organism. Although most are safe for human food and medical applications, there are monoterpene compounds that, in certain amounts or under particular circumstances (e.g., pregnancy), can cause serious disorders. The presented review characterises in vitro and in vivo, the toxic character of selected monoterpenes (α-terpinene, camphor, citral, limonene, pulegone, thujone), as well as that of their original plant sources and their essential oils. The selected monoterpenes reveal various toxic properties among which are embryotoxic, neurotoxic, allergenic and genotoxic. It is also known that the essential oils of popular plants can also reveal toxic characteristics that many people are unaware of.

## 1. Introduction

Plants have always attracted human attention. This tendency is closely connected with the pro-health properties that they hold. It is known that plants are an inexhaustible source of active compounds, namely, plant secondary metabolites. These play important roles in regulating metabolism factors, such as growth and development, protection and many other processes (i.e., symbiosis, cell signalling) in plants [1]. Moreover, their wide spectrum of biological activities ensures their use in both traditional and conventional medicine. It is worth mentioning their antioxidant and free radical scavenging activities, which are some of the most intensively studied biological activities. It is known that monoterpenes with conjugated double bonds in their structure reveal high or satisfactory activity [2]. Additionally, antifungal (i.e., thymol), antiviral (i.e., geraniol), antibacterial (i.e., myrtenal) and anti-inflammatory (i.e., pinene) activities were also observed [3].

Besides the positive influence of plant secondary metabolites, users must be aware of certain negative and harmful aspects encountered when employing natural compounds in their diet or as medicine. Such effects include errors in neurological transitions and teratogenic activity. The compounds chemically and structurally create a diverse group that is naturally present in the leaves, fruits, roots and flowers of both poisonous and non-poisonous plants [4]. The compounds can be found in various species of plant but the most popular is the *Lamiaceae* family. Among these plants are species and herbs containing a high level of monoterpenes. Among them are *Melissa* L., *Mentha* L., *Ocimum* L., and *Origanum* L., which are commonly used in everyday life [5,6]. Based on their structure, they can be classified into alkaloids, terpenes, flavonoids, phenolic compounds, resins, polypeptides, coumarins and glucosinolates. Despite comprehensive analysis of the safety of the substances for both in vitro and in vivo conditions, the issue has not been fully established. On-going research provides the latest information about various substances that were once considered safe, which now turn out to be dangerous to the body.

Among the intensively studied substances are terpenes—olatile compounds that are the most numerous group of plant secondary metabolites. The compounds have attracted the attention of researchers due to their structural differentiation and wide spectrum of biological activities [7]. However, long-lasting studies have demonstrated the harmful activity of some of these.

The review is aimed at focusing on selected monoterpenes (a subgroup of the terpenes), as well as the original plants and essential oils, which while rich in these compounds, were found to demonstrate toxic properties on the human organism. The purpose of this review is for the presentation of the side effects of selected monoterpenes and the essential oils rich in toxic properties that these compounds may then induce on the human organism at specific doses. This group of compounds was chosen due to the many pregnant women that work with these compounds and are unaware of the dangers. Most of the compounds reveal teratogenic and carcinogenic activity and can be found in everyday life.

The review is based on 81 papers on the basis of thematic consistency, the diversity of the research carried out and the availability of mechanisms of action of selected terpenes. The papers were selected using the Scopus database. The database search was based on the names of monoterpenes and their toxicity, as well as customary and Latin names for plants rich in these compounds. After conducting the comprehensive literature review, the articles were divided in terms of (1) monoterpenes; and (2) essential oils rich in these compounds. Among the papers are reviews and original articles based on in vitro and in vivo studies.

## 2. Monoterpenes: A Group of Isoprene Derivatives

**Figure 1:**
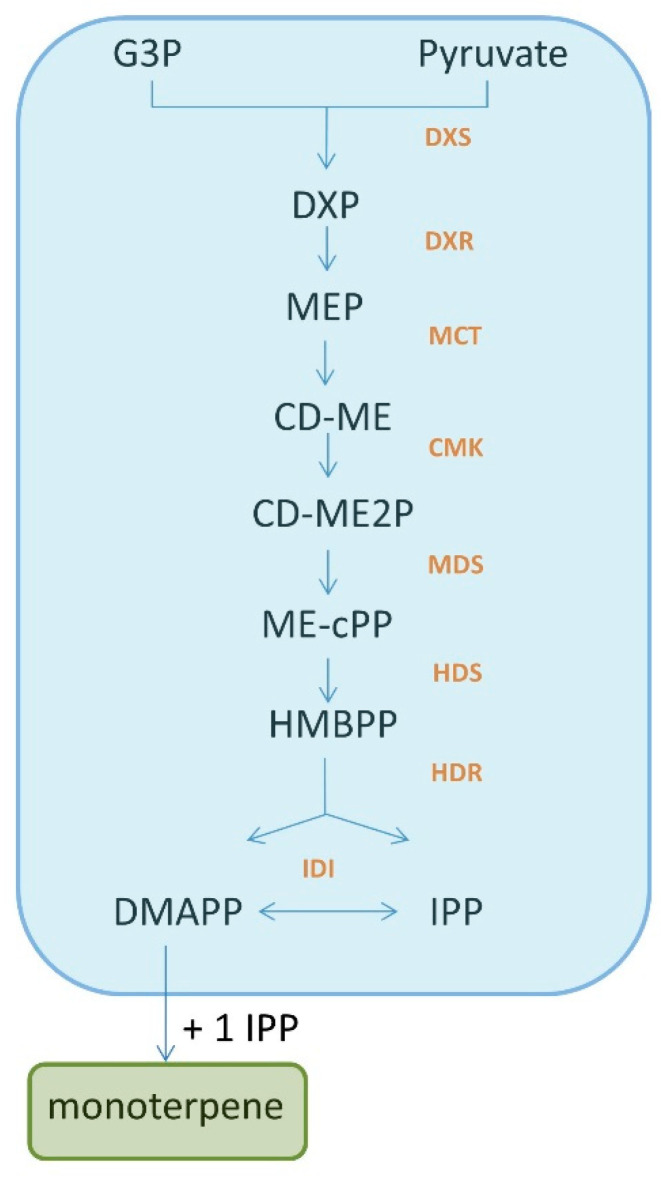
Monoterpene synthesis via IPP and DMAPP pathway based on the MEP pathway of IPP and DMAPP synthesis. The MEP pathway is built-upon pyruvate and glyceraldehyde 3-phosphate **(G3P)** condensation by thiamine diphosphate enzyme 1-deoxy-ᴅ-xylulose-5-phosphate synthase **(DXS)** to form 1-deoxy-d-xylulose 5-phosphate **(DXP)**. The latter is reduced by 1-deoxy-ᴅ-xylulose-5-phosphate reductoisomerase **(DXR)** to form MEP. In the next step, MEP is catalysed by 2-C-methyl-ᴅ-erythritol 4-phosphate cytidylyltransferase **(MCT)** to generate 4-(cytidine 5′-diphospho)-2-C-methyl-d-erythritol **(CD-ME)**. After phosphorylation, cyclisation and ring opening, CD-ME is converted into 1-hydroxy-2-methyl-2-butenyl 4-diphosphate **(HMBPP)** under the catalysis of 4-diphosphocytidyl-2-C-methyl-d-erythritol kinase **(CMK)**, 2-C-methyl-d-erythritol 2,4-cyclodiphosphate synthase **(MDS)** and 4-hydroxy-3-methylbut-2-enyldiphosphate synthase **(HDS)**, respectively. The IPP and DMAPP derived from the MEP pathway are directly generated from HMBPP by 4-hydroxy-3-methylbut-2-enyl diphosphate reductase **(HDR)**—which is different from the MVA pathway [9].

Monoterpenes are terpene isoprene derivatives. The compounds are synthesised by numerous plant families, including the *Lamiaceae* family, in response to biotic and abiotic stress, and are responsible for the aromatic character of plants. Monoterpenes can be divided, from a chemical point of view, into acyclic, monocyclic and bi- and tricyclic. Among them, the most interesting and intensively studied are the acyclic and monocyclic monoterpenes. This is due to their low molecular character [7,8]. Their biosynthesis is usually explained by the generation of dimethylallyl diphosphate (DMAPP) and its isomer, isopentyl diphosphate (IPP) through two pathways: the mevalonate pathway (MVA) and the 2-C-methyl-ᴅ-erythritol 4-phosphate pathway (MEP) [9]. MEP synthesis is shown in Figure 1.

Many of the known monoterpenes and their derivatives were studied towards their pharmacological activities. The most important of these are their antioxidant, anti-aggregatory, anti-inflammatory, anti-coagulative, sedative and analgesic properties [10,11,12]. Besides the pharmaceutical industry, monoterpenes and their derivatives find employment in the food, beverage and perfume industries. Examples include limonene, eucalyptol or linalool, which are used to generate the aroma of lemon, or 3-carene and terpinolene, which are associated with the scent of mango fruit [13,14]. Although most are safe for human food and medical applications, there are monoterpene compounds that, in certain amounts or under particular circumstances (e.g., pregnancy), can cause serious disorders.

## 3. Toxicity of Selected Monoterpenes

### 3.1. α-Terpinene

**Figure 2:**
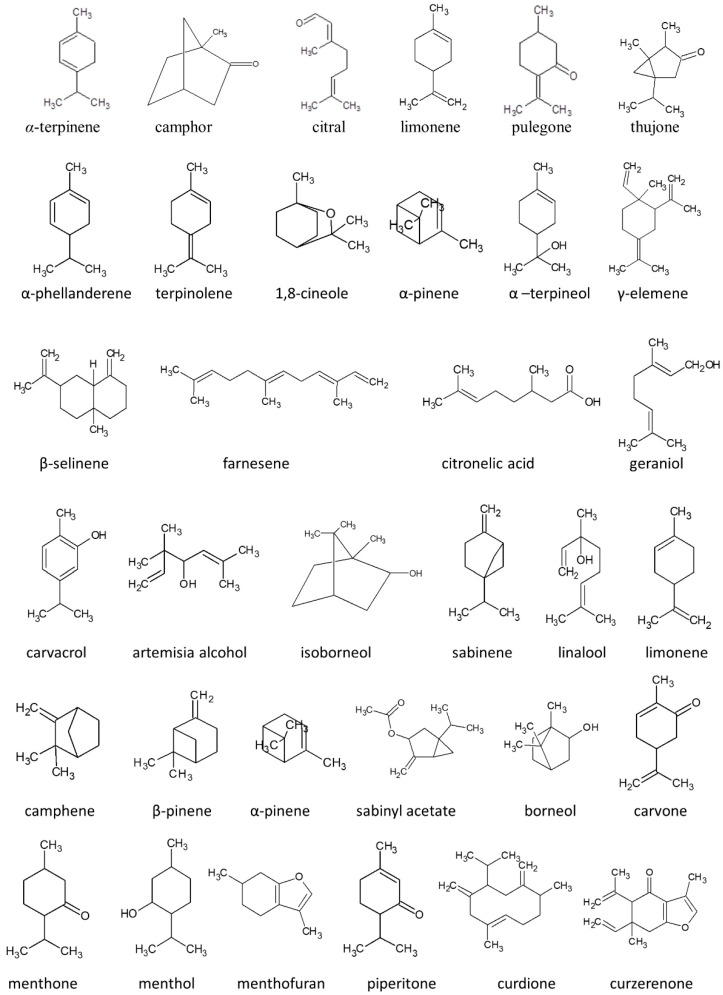
Structures of selected terpenes and their derivatives.

The common monoterpene, α-terpinene (1-isopropyl-4-methyl-1,3-cyclohexanadiene) (Figure 2), displays some rather interesting characteristics. This monoterpene can be found in numerous aromatic plants, among others, *Chenopodium ambrosidies* and *Melaleuca alternifolia* [15,16]. Research has indicated that α-terpinene demonstrates various biological and pro-health activities, such as those leading to improved neurotransmission through antioxidant or acetylcholinesterase (AChE) inhibition. Nevertheless, more and more studies, both in vitro and in vivo, have uncovered its toxic character [2,17]. Some of the first such studies were conducted by Araujo et al. and presented the embryo foetotoxic impact of the terpene in pregnant rats [18]. The studies, based on gavage administration of the monoterpene to female rats from day 6 to 15 of pregnancy, (30, 60, 125 and 250 mg/kg body weight) revealed the maternally toxic effect of 125 and 250 mg/kg body weight of α-terpinene leading to a reduction of body weight. The highest dose of the monoterpene caused a reduction of the ratio of pregnant/treated females, a decrease in foetal body weight and an increase in foetal kidney weights. Other important studies were performed by Baldissera et al. who focused on the neurotoxic effect of α-terpinene [19]. The in vivo studies, based on daily oral administration of 0.5, 0.75 and 1.0 mL kg^−1^ for 10 days, were conducted towards assessing the neurotoxic effects and activity of Na^+^, K^+^ATPase and ecto-nucleoside triphosphate diphosphohydrolase (NTPDase). The detailed analysis did not reveal depressive, anxiety or locomotor abnormalities, nevertheless, memory impairment was observed (for all dosages). The researchers noted probable neuronal DNA damage, as well as inactivation of enzymes responsible for neuronal plasticity and hydrolysis of adenosine diphosphate (ADP) and adenosine tri-phosphate (ATP). There is no precise mechanism, however, that has been discerned in explaining the neurotoxic character of the monoterpene. One possible way is decreased production of Na^+^, K^+^ATPase, which is often linked with plant product cytotoxicity [20] and is known to lead to memory and learning dysfunction [21].

Besides the neurotoxic properties of α-terpinene, it is worth mentioning the hepatic oxidative, cytotoxic and genotoxic damages brought about through monoterpene administration. Herein, valuable studies performed with the use of rat models have yielded interesting results indicating significant DNA damage and excessive generation of reactive oxygen species (ROS) in selected organs [22]. The studies were based on a rat model which was treated with α-terpinene (doses: 0.5; 0.75 and 1.0 mL/kg) (i.p) for 10 days. Liver samples were collected and assessed by histopathological analysis, caspases -1, 3, 8 assay, biomarkers of hepatic damage and determination of oxidant/antioxidant status (thiobarbituric acid-reactive substances (TBARS), catalase (CAT), superoxide dismutase (SOD), reactive oxygen species (ROS), glutathione S-transferase (GST) and glutathione peroxidase (GPx)). Significant changes in the organism were observed in all dosages. Study results revealed that each of the doses caused an increase in ROS levels and a decrease in glutathione S-transferase (GST) levels whereas 1.0 mL/kg caused an increase in thiobarbituric acids (TBARS) and glutathione peroxidase (GPx) activity. One of the important changes observed during such studies was an increase in MDA levels resulting from excessive ROS generation. This effect leads to lipid peroxidation that is harmful to the entire organism, but especially to brain function. Confirmation of excessive generation of ROS and oxidative stress development is indicated in such work through decreased (GST) activity. The enhanced activity of the enzyme, responsible for antioxidant protection, detoxification and lipid peroxidation regulation, explicitly demonstrates excessive levels of free radicals in the organism. Experiments involving rats treated with 1.0 mL kg^−1^ have also revealed increases in alanine aminotransferase and aspartate aminotransferase production. All of the studied parameters indicate that α-terpinene is able to induce oxidative stress, cytotoxic and genotoxic effects in liver tissues [22].

In accordance with the Health & Safety Executive Agency technical report (May 2021), α-terpinene should be classified as Acute Tox.4; H302 (harmful if swallowed) with an ATE of 1680 mg/kg bw. Studies based on female Wistar rats treated with α-terpinene via gavage (0, 30, 60, 125 or 250 mg/kg bw/day) between 6–15 days gestation revealed a reduction in maternal body weight (250 mg/kg bw/day). Lower doses of the monoterpene (30 and 60 mg/kg bw/d) did not affect maternal body weight gain. Additionally, delayed ossification at 60, 125 and 250 mg/kg bw/d of the monoterpene was observed. In relation to the human organism, the Committee for Risk Assessment (RAC) of the European Chemicals Agency (RAC) has appraised it, therein, in the following manner: “classification of alpha-terpinene as Acute Tox. 4; H302- Harmful if swallowed is warranted”. RAC agreed with the dossier submitter (DS, Netherlands) that classification of alpha-terpinene for acute dermal and inhalation toxicity was not warranted due to a lack of data.

### 3.2. Camphor

Camphor (2-bomanone, 2-camphonone) (Figure 2) is a bicyclic monoterpene widely used in industry. It was first derived from *Cinnamomum camphora* by distillation but currently is produced synthetically from turpentine. Camphor can be found in numerous aromatic plants, among others, *Salvia fruticosa*, *Salvia lavandulifolia*, *Santolina insularis* and *Artemisia annua* [23,24]. Camphor has been administered as a contraceptive, analeptic, cardiac, central nervous system stimulant and cold remedy, and applied as an insect repellent [25].

Studies towards its toxic properties began in the 19th century and it is known that the substance can be used medically but under specific circumstances and dosages. In accordance with available data, the lethal dose of camphor in adults ranges from 50 to 500 mg/kg. Furthermore, a dose of 2 g or more leads to toxic effects, whereas 4 *g* can result in death. In the case of children, the lethal dose is 0.5–1 g, and for infants, this is equal to 70 mg/kg [24].

Camphor can be found in the form of 20% camphor in cottonseed oil (camphorated oil) and 10% camphor in alcohol or isopropyl alcohol (camphorated spirits). Both forms are recorded as the most common poisoning agents. In accordance with OTC (Over-the-Counter) and the FDA (Food and Drug Administration), camphor-containing products cannot exceed 11% camphor. Due to these restrictions, fewer and fewer poisonings are reported [26].

Camphor poisoning is commonly recorded in children, especially in Asia, where no strict restrictions on the monoterpene exist. Poisoning, herein, can come about through ingestion or skin contact. In accordance with recent case reports, all patients needed pharmacologic intervention, and all had leucocytosis, whereas two had hyperglycemia [26]. A particular example indicative of the toxic properties of camphor is the case history of a 25-year-old Guatemalan woman who ingested a cube of the monoterpene prescribed for a facial rash. The woman used camphor resin tablets (0.25-unce) containing 99% of pure monoterpene. The solution, obtained by dissolving the tablet in 1 l of water, was drunk over 6 days. The side effects were observed within a few minutes. The oral ingestion caused headache with vertigo and disorientation, nausea, vomiting, diarrhoea, but was without eye-rolling, tongue biting and bowel incontinence [24]. Besides the prior-presented cases, there are a few case histories of other toxicosis engendered by chronic exposure to camphor. Among these, death [27] and granulomatous hepatitis [25] were recorded. The most negative influence of camphor-based treatment was observed in a child who received camphor daily, beginning at the age of 1 month as part of a remedy for fretfulness and cold. The following remedy was prepared: a 1-oz block of camphor and 8 oz of whiskey were diluted with water to a final volume of 16 oz. The total dosage of camphor given was 24.5 g or 3 g/kg thus, more than six times the amount (0.05-0.5 g/kg) known to be lethal following acute ingestion. Over a 5 months period, the infant had been given approx. 28 oz of the solution, including 4 oz during the 3 days before hospitalisation. The treatment led to the death of the infant. The death, due to camphor poisoning, is more likely the result of extensive degenerative and necrotic changes in the central nervous system limited primarily to neurons [27].

In considering the mechanisms of camphor action, its lipophilic character and ability to penetrate cell membranes should be underlined. Possible ways of absorption are transdermal, gastrointestinal tract or inhalation. It is assumed that the monoterpene is oxidised and conjugated by the liver, then excreted by the kidneys [24]. Camphor is initially metabolised via the cytochrome P450, after which it is oxidised by alcohol dehydrogenase and aldehyde dehydrogenase in the liver and conjugated with glucuronic acid to become water-soluble for urinary excretion [28]. Considering the neurotoxic properties of the monoterpene, available studies indicate nicotinic acetylcholine receptors inhibition, leading to decreasing catecholamine secretion [29].

### 3.3. Citral

Citral (3,7-dimethyl-2,6-octadienal) (Figure 2) is a mixture of two isomers: neral and geranial, which to date, has been recognised as GRAS (Generally Recognised As Safe). Due to its intensive fragrance, the volatile is commonly employed in food, cosmetics or household products. The compound has revealed a variety of biological activities (spasmolytic, analgesic, anti-inflammatory, antioxidant, diuretic), and it is often used in traditional medicine, especially in subtropical regions [30]. Indeed, the monoterpene can be found as the main constituent of *Cymbopogon citratus* [31].

Long-lasting studies have, however, revealed the negative influence of citral on the human organism. Intensive research conducted in the 1990s led to the discovery that citral, at doses above 60 mg/kg, demonstrates maternal toxicity and embryo foetotoxicity [32]. Moreover, studies performed by Duerksen-Hughes et al. have uncovered the genotoxic property of citral based on the increase in cellular p53 protein that results in DNA damage [33].

New important information about citral toxicity was presented by Souza et al., who focused on the evaluation of the genotoxic effects of the monoterpene in human cell cultures, HepG2 and leukocytes [34]. Here, different results were achieved depending on the test performed. In the case of trypan blue cell viability testing, citral showed no influence upon a decrease in leukocyte cell viability. In contrast, in the case of the MTT (3-(4,5-dimethylthiazol-2-yl)-2,5-diphenyltetrazolium bromide) assay, the monoterpene demonstrated cytotoxicity at the concentration of 5 μg/mL and above. Studies based on comet assay also indicated the significant genotoxic impact on HepG2 and leukocyte cells.

Similar study results were obtained by Sinha et al., who observed the DNA damaging potential of lemongrass (citral being its main component) [35]. Pro-DNA damage activity of citral was also noted in the case of murine peritoneal macrophages [36]. Here, the negative influence was observed at the highest concentration (100 μg/mL) and the lesions disappeared 4 h later.

In accordance with harmonised classification and labelling approved by the Europan Union, citral causes allergic skin reaction as well as skin irritation. Additionally, Registration, Evaluation, Authorisation and restriction of Chemicals (REACH) registrations indicated serious eye irritation by the compound [37]. In accordance with the Screening Information Dataset (SIDS) Initial Assessment Report for the 13th SIAM (2001), underlining the high volatility of citral which causes rapid loss from the skin but the remaining amount of the terpene is well absorbed. Scientists underlined the low acute toxicity of the compound in rodents in comparison to humans. Available data of citral toxicity were summarised by Api et al. in “RIFM fragrance ingredient safety assessment, citral, CAS Registry Number 5392-40-5*”* [38]. Based on various study results, scientists present citral as a substance without concern for genotoxic potential whereas the total systematic exposure to citral (7.2 µg/kg/day) is below the Threshold of Toxicological Concern (TTC) (30 µg/kg/day) for the repeated dose toxicity endpoint of a Cramer Class I material at the current level of use [39].

### 3.4. Limonene

Limonene (1-methyl-4-(1-methylethenyl)cyclohexane) (Figure 2) is a monocyclic monoterpene that is the main component of various citrus oils (orange, lemon, lime and grapefruit) [37]. Limonene is characterised by low polarity. This limits its cell membrane passage and causes less activity in in vivo conditions [40]. In mammals, limonene, after absorption, is rapidly distributed within various tissues and metabolised into other active compounds. Among these, the most important are peryllic acid, dihydroperyllic acid and limonene-1,2-diol (the main metabolites), but 1,2- and 8,9-epoxide, carveol and perilyl alcohol were also found in some animal species [40,41]. Half-time of limonene ranges from 12 to 24 h and it can be excreted (83%) in the urine within 48 h [39].

The monoterpene is recognised as GRAS, thus, it is often used as a flavouring agent in food, cosmetics and household products. Nevertheless, long-lasting studies have revealed some negative influences on the mammalian organism. The following lethal doses (LD50) for limonene are known: male and female mice: 5.6 and 6.6 (oral administration), 1.3 and 1.3 (intraperitoneal administration), >42 (subcutaneous administration) g/kg body weight, respectively; male and female rats: 4.4 and 5.1 (oral administration), 3.6 and 4.5 (intraperitoneal administration), >20 (subcutaneous administration) g/kg body weight, respectively [42].

In vivo studies based on mouse and rat models have revealed that the monoterpene has an impact on cancer development and that it holds allergenic or sensitising potential [41]. Herein, intensive research in the 1990s demonstrated the development of kidney cancer in male rats after exposure to limonene. Nevertheless, a detailed analysis of limonene’s mechanism of action revealed that the toxicity is not relevant to humans [43]. Other studies have indicated that limonene administration affects uridine diphosphoglucuronosyl transferase and total cytochrome P450 enzymes [44]. Additionally, limonene above concentrations of 100 μM was shown to be highly toxic to human lung cells [41].

Considering reproductive toxicity, teratogenic or embryotoxic effects were not observed after limonene exposure. Nevertheless, the compound may indirectly affect fertility by inducing decreased body weight and growth in mothers. This effect leads to a pivotal increase in skeletal anomalies and delayed ossification in their offspring [42]. Moreover, in rabbit models, studies have revealed that high doses of limonene can reduce weight gain in dams, and in some such amounts, lead to death [42].

In the case of clinical management, various side effects of limonene exposure were observed. The most common is skin sensitisation. In addition, burning pains in the mouth and throat, nausea, vomiting, ataxia, coughing, choking, fever and tachycardia have occurred after monoterpene ingestion [42].

Similar to citral, Registration, Evaluation, Authorisation and restriction of Chemicals (REACH) presented registrations based on limonene toxicity. In accordance with the harmonised classification and labelling approved by the European Union, the monoterpene is very toxic for aquatic life with long-lasting effects as well as causing skin irritation and allergic skin reaction. Additionally, limonene can be fatal if it is swallowed and enters the airways [45].

### 3.5. Pulegone

Pulegone (2-isopropylidene-5-methylcyclohexanone) (Figure 2) is a colourless monoterpene ketone that can be found in the essential oils of various plants, especially mint species (i.e.*, Hedeoma pulegoides*, pennyroyal and *Nepeta cataria)* [46]. Similar to other monoterpenes, pulegone displays a variety of biological activities confirmed in in vitro, in vivo and ex vivo studies. Among the most important are the following: antioxidant (in vitro spectrophotometric studies revealed that pulegone is able to scavenge over 90% of free radicals at a concentration equal to 0.8 M within 5 min) [2]; anti-inflammatory (inhibition of lens protein-induced inflammation by 58% when 50 µL of 0.5% pulegone was instilled into the cul de sac of rabbit eyes) [47]; and antihyperalgesic effects (in vivo studies confirmed pulegone a transient antihyperalgesic effect on both mechanical, thermal heat and cold at a dosage of 100 mg/kg) [48]. Despite many cases of human poisoning, the compounds are often used in herbal medicine and in the cosmetics and food industries [49].

**Figure 3:**
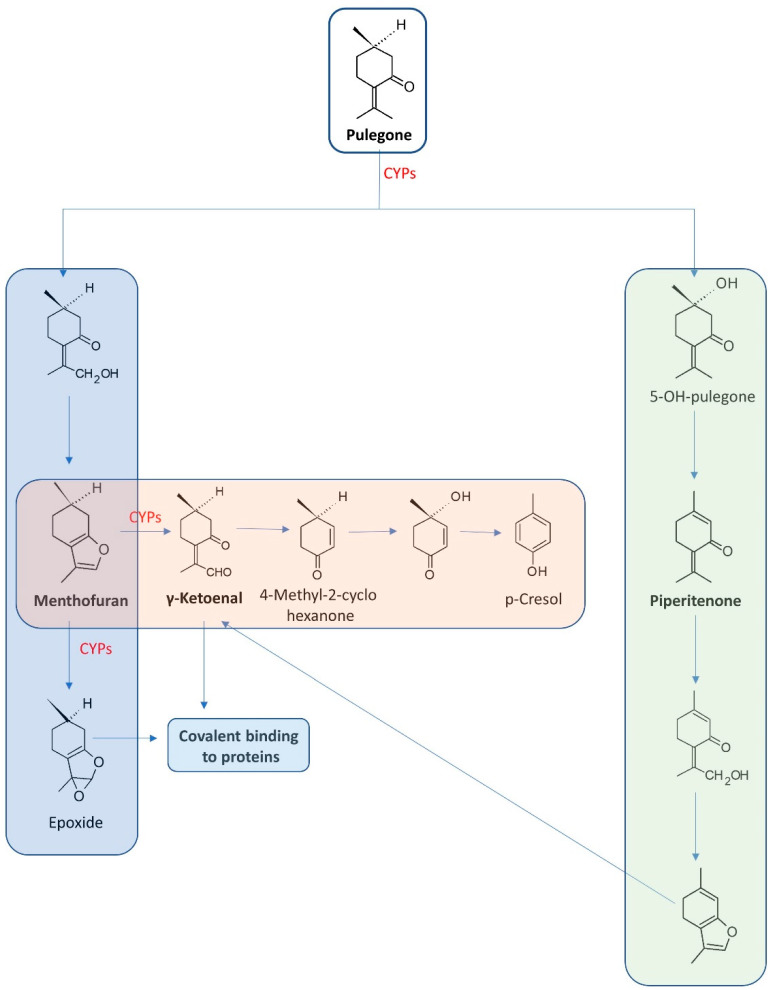
Metabolism of pulegone. The most important metabolites are emboldened.

Long-lasting studies have enabled the determination of pulegone’s metabolites. Under in vivo conditions and via rat models, three main metabolic pathways were uncovered: 1. Menthofuran formation; 2. Formation of piperitenone, and 3. Conjugation by glucuronic acid following C-5 or methyl group hydroxylation or by GSH [50]. Oral consumption of the compound leads to its metabolisation into 14 metabolites, among which, menthofuran and 8-pulegone aldehyde are more toxic than pulegone. Within the metabolites, γ-ketoenal is characterised as a major reactive electrophilic metabolite. It is also highly probable that the compounds can create covalent bonds with cellular proteins in the liver—leading to its injury [51]. Moreover, the metabolites of pulegone have revealed differing toxicity levels [50,52]. Figure 3 presents the metabolic activation of pulegone.

In assessing the toxicity of pulegone, menthofuran and p-cresol are known as naturally occurring hepatotoxin- and glutathione-depleting agents, respectively. Significant examples of the hepatotoxic effect of pulegone and menthofuran are hepatomegaly, poor perfusion and dark blood from the nasogastric tube and rectum. The changes were observed for serum concentration of pulegone at 25 ng/mL and menthofuran at 41 ng/mL [53]. A major electrophilic metabolite is γ-ketoenal. This is generated from both pulegone and menthofuran. 

Studies performed on the mice model have revealed poor liver function, increased liver and kidney weights, nasal epithelia and atrophy of female reproductive organs as a consequence of p-cresol administration at doses of 30,000 ppm in the diet [54]. Although the majority of the scientific reports on pulegone toxicity relate to rodent models, valuable results were obtained for research based on the human livers of five patients. Detailed analysis therein indicated pulegone as being more hepatotoxic than menthofuran. In contrast, the mice and rat models have revealed the higher toxicity of menthofuran. Additionally, pulegone was found to be able to change the expression of certain miRNAs in liver samples [55].

In accordance with the “Public statement on the use of herbal medicinal products containing pulegone and menthofuran” (2016), tolerable daily intake (TDI) for pulegone was set for food (0.1 mg/kg bw) and doses up to ca 2.3 mg/kg bw/day (exceeding the TDI for food) [56]. In cosmetic formulations, the concentration of pulegone should not exceed 1%. In the USA, the monoterpene is not authorised as a synthetic flavouring substance (DHHS-FDA, 2012) [57].

### 3.6. Thujone

Thujone, a bicyclic monoterpene ((1S,4R,5R)-1-Isopropyl-4-methylbicyclo [3.1.0]hexan-3-one) (Figure 2) naturally occurs as α-thujone and β-thujon—two enantiomeric forms. The monoterpene is widespread in nature and can be found in various plants and essential oils, among which the most important is *Artemisia absinthium* and *Salvia officinalis* [57,58]. Thujone has been intensively studied. The phenomenon results from the fact that the compound is often used to flavour foods and beverages, and is a significant constituent of the wormwood-flavoured spirit, absinthe. Ingestion of the alcohol cause symptoms of so-called “absinthism” characterised by convulsions, blindness, hallucinations and mental deterioration [59,60]. Due to the neurotoxic character of thujone, in most countries, there are regulations regarding the amount of terpene allowed in food and beverages. Nevertheless, some herbs containing thujone are recognised by the Food and Drug Administration as generally safe (see: Food and Drug Administration (FDA), 2010. Listing of Food Additive Status Part I.). In accordance with the European Medicines Agency (EMA) regulations [61] contained in the *Artemisia absinthium* monograph, the daily intake should not exceed 3 mg/person/day for a maximum duration of 2 weeks.

Long-lasting studies based on animal models, cultured neuronal cells and expressed receptors towards thujone neurotoxicity have allowed an understanding of its mechanism of action. It is known that both enantiomers are able to modulate γ-aminobutyric acid (GABA-gated) chloride channels, but the isomer α is two- to three-fold more potent than isomer β. The cannabinoid CB 1 and serotonin 5-HB3 receptors are additional targets for thujone activity. However, research indicates that the neuronal effect is completely reversible [62,63]. Thujone displays rapid absorption due to its small molecular weight and lipophilic character. Its precipitate metabolic processing leads to creating 7-hydroxy-(α-thujone) as the main metabolite [62].

There are a limited number of studies available on the metabolic results of thujone ingestion. Examples are studies performed by Siveen and Kuttan [64]. The basis of them were 6-8-week-old mice divided into five groups. The control group received paraffin oil while the experimental group received thujone (1, 5, 10 and 25 mg/kg/bw) intraperitoneally for 14 days. Study results showed behaviour changes, enhanced mortality, dissection out and decrease in organ weight (liver, spleen, thymus, kidney and lungs), as well as disturbances in hepatic and renal functions which were observed for a 25 mg/kg dose, whereas a dose of 1 mg/kg was selected as non-toxic. The greatest change was observed for the weight of the liver for which a decrease in weight from 5.64 g/100 g b.w. to 4.72 g/100 g b.w. was noted [64]. Other studies based on male and female rats and mice investigated the toxicokinetics of thujone [65]. The studies based on gavage administration of α-thujone or a mixture of α- and β-thujone were performed with the following parameters: for single gavage administration: male and female rats and mice were given a single gavage dose of α-thujone or a mixture of isomers at doses of 25 or 50 (rats) or 40 or 80 (mice) mg/kg whereas for intravenous administration the following parameters were used: a single intravenous dose of 1.6 (rats) or 3.2 (mice) mg/kg of α-thujone and 3 (rats) or 6 (mice) mg/kg of a mixture of isomers. Obtained results have revealed that elimination of α-thujone in the brain was slower than in the plasma. This effect can impact the neurotoxic effect of the terpene. It was also observed that in rats the compound was more easily absorbed in females than males. This difference, however, was not observed in mice [65]. Beyond its neurotoxicity, the compound reveals other negative properties, such as genotoxicity and carcinogenicity [66].

In accordance with the “Public statement on the use of herbal medicinal products containing thujone” (2011), the daily intakes of thujone in form of *Absinthii* herba was set at 3.5 mg/person, and in the case of sage leaf preparations at 5.0 mg/person, both for a maximum duration of 2 weeks [67]. Thujone is not authorised for use as a flavouring substance in the USA, whereas in France and the United Kingdom the mean daily intake of thujone is estimated to be between 0.27 and 1.09 mg/person [68].

The toxicity of the selected monoterpenes is summarised in Table 1.

## 4. Plants and Their Essential Oils Rich in Monoterpenes

Many plant families are rich in monoterpenes. Most are recognised as safe, but an overdose of their essential oils can be harmful to humans.

It is known that monoterpenes can be found in herbs and species commonly used in everyday life [5,6]. Usage of the plants can cause allergic reactions or medical accidents. Particular attention should be paid to them by pregnant women due to the fact that a significant number of terpenes reveal teratogenic toxicity or changes in reproductive organs [54]. A characteristic feature of terpenes is their aromatic character which results in essential oils consisting of these compounds being commonly used in cosmetics and household products. Among them, very popular are lemongrass, verbena, lavandula or citrus fruits essential oils [68]. Considering their aromatic qualities, it is impossible not to agree with their common use. However, are too high a concentration and the frequent use of these oils harmful to the body?

Table 2 provides a listing of plant sources of monoterpenes, along with a qualitative analysis of their essential oils and the toxicity observed in various types of studies.

## 5. Conclusions

The review was focused on the toxic properties of selected monoterpenes that are well known and often used in various industries including the food and pharmaceutical industries. It is recognised that most of the known monoterpenes reveal valuable biological activities, but some of them also demonstrate a toxic character. Their negative influence upon the human organism has to be underlined in the case of their usage in food or medicine. Among the presented compounds, the most toxic are thujone, camphor and pulegone. These have a neurotoxic, genotoxic or teratogenic impact on the organism. Additionally, essential oils rich in monoterpenes have shown a toxic character evaluated in in vitro and in vivo studies, thus their usage should be closely controlled. Unfortunately, in some cases, the side effects of monoterpenes may outweigh the benefits.

In future studies, the determination of the influence of selected monoterpenes on coordination and memory/learning enhancement in mice models will be focused on. Among them, there will be no compounds that are toxic to humans.

## Figures and Tables

**Table 1:** Toxicity of selected monoterpenes.

Monoterpene	Toxicity	Type of Study	References
α-Terpinene	Embryo foetotoxicity	In vivo—rats model	[18]
	neurotoxic	In vivo—rats model	[19]
	memory and learning dysfunction	In vivo—mice model	[21]
	hepatic oxidative cytotoxic genotoxic	In vivo—rats model	[22]
	lipid peroxidation	In vivo—rats model	[22]
Camphor	neurotoxic	In vivo—human case report	[24]
	hepatotoxicity granulomatous hepatitis	In vivo—human case report	[25]
Citral	Embryo foetotoxicity maternal toxicity	In vivo—rats model	[32]
	genotoxic/DNA damage	Cultured cells	[33]
	genotoxic/HepG2/leukocytes	Cultured cells	[34]
	DNA damage	human lymphocytes	[35]
Limonene	cancerogenic allergenic sensitising	In vivo—mice and rats model	[41]
	toxic for human lung cells	Human lung cell culture	[41]
	impact on uridine diphosphoglucuronosyl transferase	Human cell culture	[44]
Pulegone and its metabolites p-cresol and menthofuran	hepatotoxin nasal epithelia atrophy of female reproductive organs	In vivo—mice models	[51]
	hepatotoxic change in expression of miRNAs	Human liver	[55]
Thujone	neurotoxic/modulation of GABA-gated chloride channels	In vivo—mice and rats models In vivo—human liver preparations	[62,63]
	decrease in organ weight behaviour changes disturbances in hepatic and renal functions	In vivo—mice models	[64]
	genotoxicity carcinogenicity	Bacteria and mammalian cells	[64]

**Table 2:** Examples of plants and their essential oils rich in monoterpenes and their potential toxic character. Structures of the main components are presented in Figure 3.

Plant/Essential Oil	Oil Composition *	Hazards	Commentary	References
*Eucalyptus staigeriana* F. v. Muell. ex F. M. Bailey	d-limonene+ α-phellandrene (30.5%), **geranial (9.9%), neral** **(7.7%)**, and terpinolene (6.6%)	Teratogenicity (malformation and abnormal eye development)	Maximum oral dose in pregnancy: 238 mg/day	[69]
*Vitex agnus-castus*	Leaf EO: 1,8-cineole (15.6–35.2%), sabinene (6.9–17.1%), α-pinene (1.0–13.9%), α-terpineol (1.4–9.2%), γ-elemene (0–9.1%), β-selinene (0–9.0%), β-caryophyllene (2.3–8.9%), (Z)-β-farnesene (0–8.6%), citronellyl acetate (0.3–7.8%), and citronellic acid (0–6.6%), **may contain methyleugenol**	Reproductive hormone modulation (lower prolactin levels and prolongation of menstrual phase)	The component that is probably responsible for the side effect is methyleugenol. Study results support the use of 20 mg VAC dry extract, ethanol 60% m/m, daily for treatment of PMS	[70,71]
*Thymus citriodorus* (Pers.) Schreb. (Synonyms: *Thymus lanuginosus* Mill. var. citriodorum Pers., *Thymus serpyllum* var. citriodorus (Hort.), *Thymus* *serpyllum* var. *vulgaris* Benth.); a cross between *Thymus vulgaris* and *Thymus pulegioides*.	geraniol (39.2%), carvacrol (15.4%), **geranial (9.2%) and neral** **(7.1%)**	Teratogenicity (changes based on oxidation processes and effects on proliferation level)	Maximum oral dose in pregnancy: 258 mg/day.	[69,72]
*Backhousia citriodora* F. Muell.	**geranial (46.1–60.7%) and neral** **(32.0–40.9%)**	Teratogenicity (dose-dependent malformations in chicken embryos; fetal cranial development)	Maximum oral dose in pregnancy: 46 mg/day.	[73,74]
*Artemisia vulgaris*	camphor (20.8%), artemisia alcohol (15.3%), **α-thujone** **(11.4%)**, β-caryophyllene (10.6%), isoborneol (9.3%), 1,8-cineole (9.0%), and sabinene (6.1%)	Neurotoxic (due to -aminobutyric acid type A (GABAA) receptor modulation of thujone)	Thujone was revealed as having the highest neurotoxic activity within the plant and is considered as being responsible for its neurotoxic character.	[75]
*Salvia lavandulifolia* Vahl	1,8-cineole (12.0–40.3%), camphor (12.9–36.1%), α-terpinyl acetate (0.5–15.5%), linalool (0.2–11.2%), α-pinene (4.7–10.9%), camphene (4.6–10.6%), β-pinene (3.3–7.3%), **(Z)-sabinyl** **acetate (0.5–9.0%)**, borneol (1.5–6.4%), linalyl acetate (0.1–5.8%), and limonene (2.4–5.0%)	abortifacient (reduction of maternal weight leading to abortifacient effect), teratogenicity	Studies performed on mice have demonstrated teratogenicity and dose-dependent abortifacient effects of sabinyl acetate. Sage is contra-indicated during pregnancy and lactation.	[76,77]
*Artemisia herba-alba* Asso	camphor (34.0–55.0%), **α-thujone** **(25.7–36.8%), β-thujone** **(2.0–9.0%)**, camphene (0.5–9.0%), and 1,8-cineole (1.5–8.0%)	Neurotoxic (due to -aminobutyric acid type A (GABAA) receptor modulation of thujone)	The essential oil is recognised as neurotoxic due to contained high levels of thujone.	[78,79]
*Mentha piperita*	menthol (29–48%), menthone (20–31%), **menthofuran (6–8%), pulegone,** menthyl acetate (3–10%), limonene, pinene and piperitone	Nephropathy (hyaline droplet formation), carcinogenic effect (hepatocellular carcinoma at higher doses)	This side effect was observed in rats after subchronic administration of peppermint oil (100 mg/kg/day). The essential oil is able to exchange human lymphocytes and induce chromosomal aberrations. It is to ensure that the sum of pulegone and menthofuran within the daily dose is < 37.5 mf for adults. To reach the limits, peppermint oil with adequate quality (specification of adequate limits of pulegone and menthofuran) is required.	[80,81]
*Mentha spicata*	piperetenone oxide (49.4%), carvone (15.3%), 1,8-cineole (5.8%) and **limonene (2.7%)**	Uterine damage (caused by apoptosis and diffuse eosinophil leucocyte infiltration in surface and stromal glandular epithelium in both endometrium and endocervix strictly connected with lipid peroxidation)	Histopathological changes such as apoptosis and diffuse eosinophil leucocyte infiltration were observed in rats administered *M. spicata* tea. To reach the limits, peppermint oil with adequate quality (specification of adequate limits of pulegone and menthofuran) is required. As A general precaution, it is not recommended to use *M. spicata* during pregnancy and lactation, unless medical advice proposed benefit is higher than the potential risk.	[82,83]
*Curcuma zedoaria* Roscoe	epicurzerene (19.0–46.6%), curzerene (10.4%), curdione (7.0–19.6%), curzerenone (22.3–31.6%), debromofiliforminol (31.5%), 1,8-cineole (18.5–40.8%), β-sesquiphellandrene (21.5%), p-cymene (18.4%), curcumenene (18.7%), and α-phellandrene (14.9%)	antigestational embryotoxicity, antifertility; and abortifacient (strictly connected with decreased body weight of maternal; embryonic angiogenesis inhibition was observed)	The component responsible for the side effects has not been identified. Reproductive toxicity observed in mice treated with up to 10 g/kg/day water extraction of the plant.	[84]

* the most toxic components are bolded.

## Data Availability

Not applicable.
